# 

**Published:** 1905-08

**Authors:** 


### LITERARY NOTE.

Cunningham's Anatomy.—Messrs. William Wood & Company announce a forthcoming new edition of Cunningham's Textbook of Anatomy. During the two years of this book's existence, it has sprung into universal favor and is now the standard text-book in a majority of the prominent medical schools of this country.

